# Lamb-dip spectroscopy of the C−N stretching band of methylamine by using frequency-tunable microwave sidebands of CO_2_ laser lines

**DOI:** 10.1038/srep34270

**Published:** 2016-09-29

**Authors:** Zhen-Dong Sun, Shan-Dong Qi, Ronald M. Lees, Li-Hong Xu

**Affiliations:** 1 School of Physics, Shandong University, Jinan 250100, School of Physics and Electrical Engineering, Kashgar University, Kashi 844006, P. R. China; 2 Department of Physics, University of New Brunswick, Saint John, New Brunswick E2L 4L5, Canada

## Abstract

Lamb-dip spectroscopy of the C−N stretching band of methylamine has been systematically extended to *P*-, *Q*-, and *R*-branch by using microwave sidebands of a large number of CO_2_ laser lines as frequency-tunable infrared sources in a sub-Doppler spectrometer. Lamb-dip signals of more than 150 spectral lines have been observed with a resolution of 0.4 MHz and their frequencies have been precisely measured with an accuracy of ±0.1 MHz. More than 30 closed combination loops have been formed, which unambiguously confirm the assignments. For over 150 vibrational excited levels in 27 substates, refined term values have been determined and expanded in *J*(*J* + 1) power-series to determine the substate origins and the effective rotational constants. For transitions with *Aa* torsion-inversion symmetry in torsional state *υ*_t_ = 0, 57 *K*-doublet lines displaying asymmetry splittings have been observed and the splitting constants for levels with *K* = 1, 2, and 3 in the excited states have been determined. Our results provide accurate experimental information for spectroscopic studies of the interesting vibrational perturbations and intermode interactions related to the C−N stretching mode, directly support astronomical surveys, and are very relevant in practice to identification and frequency determination of the CO_2_-laser-pumped far-infrared laser lines of methylamine.

Methylamine (CH_3_NH_2_) is the simplest primary amine in chemistry. Its CH_3_ group is connected to the NH_2_ group by the C−N bond that exhibits a vibrational stretching motion. As for the two groups themselves, the CH_3_ group has an internal torsional motion while the NH_2_ group has an inversion motion, which makes the CH_3_NH_2_ a prototype in molecular physics for non-rigid molecules having two coupled large-amplitude internal motions. The torsion splits the rotational energy levels into a threefold pattern of *E* and *A* symmetry components with an *E*/*A* spin statistical weight ratio of 1/2, and inversion produces a further splitting into *s* and *a* doublets with spin weight ratio of 1/3. Thus, each vibration-rotation level of CH_3_NH_2_ has *Aa, As, Ea* and *Es* sublevels, resulting in a complex vibration-rotation-torsion-inversion energy structures and rich but highly crowded spectra with a wide range of relative line intensities. Therefore, the interesting microwave^1^,^2^,^3^,^4^,^5^,^6^,^7^,^8^,^9^,^10^, far-infrared^11^,^12^,^13^,^14^,^15^,^16^,^17^, and infrared^18^,^19^,^20^,^21^,^22^,^23^ spectra of CH_3_NH_2_ have been extensively studied for many years to explore the splittings and the symmetry species, leading to the valuable application in the detection of interstellar methylamine^24^,^25^,^26^,^27^. In contrast, despite the fact that it is of great practical interest related to optically pumped far-infrared laser lines and interstellar detection, spectroscopic study by the sub-Doppler technique on the stretching of the C−N bond that connects the CH_3_ and NH_2_ groups has remained rare until recently^28^.

Study of the C−N stretching band of CH_3_NH_2_ has been of great spectroscopic, practical, and astrophysical interest for decades. The C−N stretching band of CH_3_NH_2_, earlier studied and reported sixty years ago at the relatively low resolution of 1 cm^−1^ and an accuracy of about 0.1 cm^−1^ by Gray and Lord^19^, displays strong characteristic absorptions in the infrared region around 1044 cm^−1^. It is characterized by a parallel structure with vibrational *P, Q*, and *R* branches, which overlaps well with the CO_2_ laser bands. Thus, CH_3_NH_2_ has been an important member of the class of laser media employed to generate far-infrared laser lines by optically pumping with CO_2_ laser transitions. However, few of the CH_3_NH_2_ lines have so far been identified as to their quantum numbers due to the limited accurate experimental information available on this important band. It is only recently that high resolution spectroscopy of the C−N stretching band of CH_3_NH_2_ has been reported. In 2011, Lees *et al*.^21^ and Gulaczyk *et al*.^22^ investigated Doppler-limited Fourier-transform (FT) spectra of this band. Numerous transitions were assigned, perturbations from several resonances between the C−N stretching state and high-lying torsional substates of the ground state were analyzed, with both Fermi and Coriolis resonances being observed. Yet to date there are still many overlapped lines and unresolved asymmetry doublet lines in the FT spectrum for this band of CH_3_NH_2_ due to limit on spectral resolution imposed by the Doppler width. It is very desirable to carry out sub-Doppler observations on this band employing a spectrometer with very high resolution and accuracy in order to make confident and reliable measurements and line assignments. In 2010, we observed the precise Lamb-dip spectra of the C−N stretching band of CH_3_NH_2_ at a spectral resolution of 0.4 MHz and determined the transition frequencies with an accuracy of ±0.1 MHz^28^, which is the first sub-Doppler observation in any spectral range of CH_3_NH_2_. However, the first Lamb-dip spectra of this band have been observed just for 43 saturation dips which are primarily in the C−N stretching *Q*-branch region and only in two sub-states. There is a clear need to observe many more Lamb-dip signals over a wider range of transitions in *P*-, *Q*-, and *R*-branch to obtain accurate experimental information and parameters for detailed spectroscopic analysis. These precise Lamb-dip measurements are important not only for disentangling overlapped features in the Doppler-limited spectra but also in providing a grid of standard reference frequencies for accurate calibration of the overall FT spectrum. Accordingly, in the present work, using frequency-tunable microwave sidebands of a much larger group of CO_2_ laser lines in our spectrometer^28^, the Lamb-dip spectroscopy of the C−N stretching band of CH_3_NH_2_ has been systematically studied to cover a greatly expanded set of quantum states. We now report our extended experimental results.

## Results and Discussion

### A frequency-tunable infrared source for Lamb-dip observation

**Figure 1:**
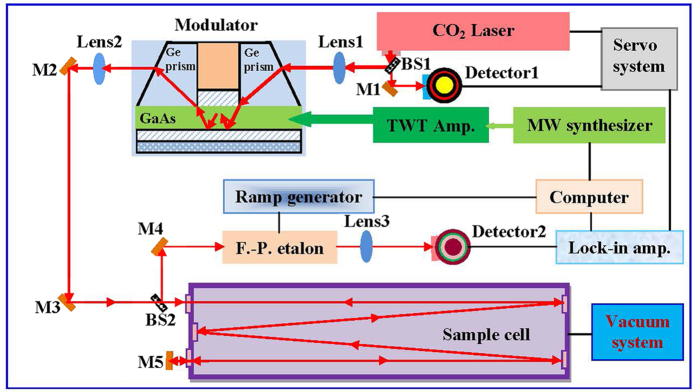
Schematic of the sub-Doppler spectrometer using microwave sidebands of CO_2_ laser lines as frequency-tunable infrared sources for the Lamb-dip spectroscopy of CH_3_NH_2_. M1–M5, mirrors; BS1, BS2, beam splitters; MW, microwave; TWT Amp., traveling wave tube amplifier; F.–P. etalon, Fabry–Pérot etalon filter; Detector1, InSb detector; Detector2, HgCdTe detector.

Although the C−N stretching band center of CH_3_NH_2_ overlaps well with the CO_2_ laser bands, it is difficult to observe many absorption lines of CH_3_NH_2_ by using just a grating CO_2_ laser, because the available spectral coverage between the CO_2_ laser and CH_3_NH_2_ absorption lines is limited to the overlap in Doppler widths of only about 60–100 MHz for each laser line. This situation was recently improved greatly by the application of a frequency-tunable infrared source in a dual-mode sideband spectrometer to CH_3_NH_2_^28^. The schematic of the experimental setup used in the present work for the Lamb-dip spectroscopy of CH_3_NH_2_ is shown in Fig. 1, and has been described in detail previously^28^. The tunable radiation is generated by adding microwave sidebands to the CO_2_ laser lines in a GaAs waveguide modulator. In the modulator, the added microwave radiation with a frequency *f*_MW_ produces a periodic variation of the refractive index of the GaAs crystal and a corresponding small phase change of *Δϕ* to the incident CO_2_ laser field. Therefore, the laser output from the modulator includes both the input field of the CO_2_ laser plus microwave-modulated field components. The latter are called microwave sidebands of the CO_2_ laser lines. In practice, the output from the modulator shows three spectral signals at *f*_L_ (frequency of CO_2_ laser carrier), *f*_L_ + *f*_MW_ (upper sideband), and *f*_L_−*f*_MW_ (lower sideband), respectively. These upper and lower microwave sidebands of CO_2_ laser lines provide a powerful radiation source for precision spectroscopy, operating at room-temperature with narrow linewidths and continuously tunable and precisely controlled frequencies in the 9–11 *μ*m region. Our infrared source has the three main features of 1) a tunability range about 23.6 GHz (±6.7 to ±18.5 GHz) for each CO_2_ laser line by sweeping the microwave frequency *f*_MW_; 2) a typical microwave-modulated CO_2_ laser power about 10 mW for either the upper or lower sideband; 3) accurate radiation frequency. In operation, the CO_2_ laser is stabilized to the center of a 4.3 *μ*m fluorescence Lamb-dip signal and has an estimated frequency uncertainty of 33 kHz^29^.

### High-resolution observation and precise measurement of the spectral Lamb-dips

**Figure 2:**
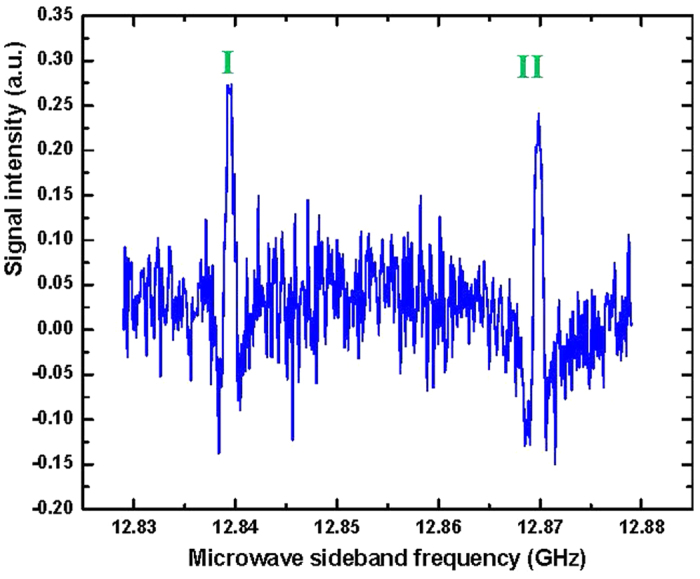
Observation of two Lamb-dip signals I and II resolved from an overlapped feature in the FT spectrum of CH_3_NH_2_. The spectrum was recorded in a scanning range of 50 MHz using the lower microwave sideband of the 9*P*22 CO_2_ laser line. The sample pressure was 20 mTorr and the lock-in time constant was 30 ms. Signals I and II are assigned as transitions *Q*(0 *As* 8, 8) and *Q*(0 *As* 5, 5), respectively. The observation of Signal II has previously been reported in Ref. 28 and has been confirmed here. This chart demonstrates features of the high power for observation of the saturated absorption spectra and the wide frequency-tunability of the microwave sidebands of CO_2_ laser lines.

**Figure 3:**
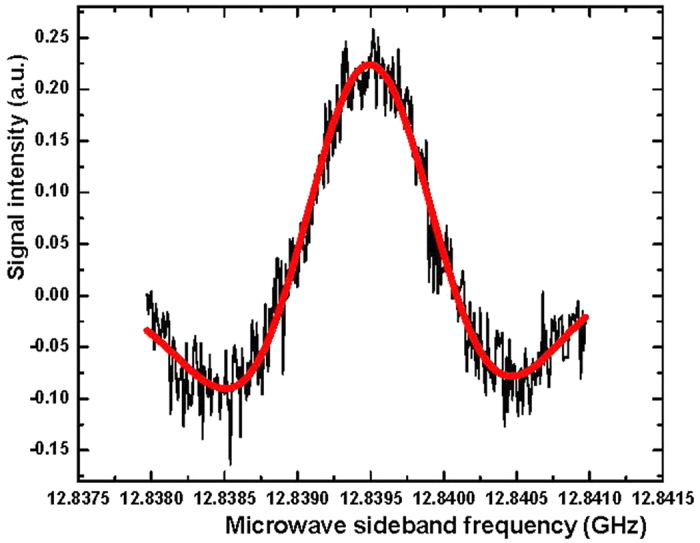
Lamb-dip signal for the *Q*(0 *As* 8, 8) line of the C−N stretching band of CH_3_NH_2_ (signal I in Fig. 2), scanned in a range of 3 MHz with a step-size of 10 kHz and recorded with the lock-in amplifier in 2*f* detection mode. The lock-in time constant was 30ms and the sample pressure of CH_3_NH_2_ was 14 mTorr. The solid curve (red) is a least-squares fit of a second-derivative Gaussian profile to the experimental trace (black). The microwave frequency at line center was determined from the fit to be 12839.477 MHz.

A Lamb-dip signal has a much narrower spectral linewidth than that of the Doppler-broadened spectral line. The Lamb-dip spectroscopic technique can thus enable blended lines in the Doppler-limited spectrum to be fully resolved and the centers of these resolved absorption lines to be determined very precisely. Figure 2 shows a Lamb-dip spectrum for a blended line in the extremely congested *Q*-branch of the C−N stretching band center of CH_3_NH_2_ around 1044.5930 cm^−1^ in the FT spectrum. A 50 MHz scan of the lower microwave sideband of the 9*P*22 CO_2_ laser line was recorded at a pressure of 10 mTorr in 2nd derivative (2*f*) detection mode using a lock-in amplifier time constant of 30 ms. We can see that two spectral lines with an interval of just 30 MHz have been clearly resolved. This shows the usefulness of the high power and wide frequency-tunability of the CO_2_-microwave sidebands for resolving the overlapped features by Lamb-dip observations. Furthermore, in order to precisely measure the absolute transition frequencies for each of the individual line, we narrowed the microwave scanning range down to 3 MHz and swept the microwave sideband both upward and downward 5 times for each line with a frequency step-size of 10 kHz to record their saturation-dip 2*f* signals. Figure 3 displays the result for signal I of Fig. 2, recorded at 14 mTorr pressure with a lock-in time constant of 30 ms. We then fitted the experimental trace (black) to determine the center transition frequency by a least-squares fit to the second derivative of a Gaussian profile^29^


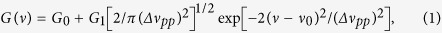


where *G*_0_ is a baseline constant, *G*_1_ is the integrated intensity, ν_0_ is the center frequency, and *Δ*ν_*pp*_ is the frequency separation between positive and negative peaks of the first-derivative *G*’(ν). The fitting trace is shown as a red solid curve in Fig. 3. From this fit, the microwave frequency at line center was determined to be 12839.477 MHz, which yields an infrared transition frequency as 9*P*22–12839.477 MHz, giving 31 316 122.019 MHz with an accuracy of 0.1 MHz when the known frequency of the 9*P*22 CO_2_ laser line^30^ is added. In the current study, we have measured more than 150 saturated absorption dips for spectral lines which belong to 27 C−N stretching substates. The assignments of these transitions, the measured transition frequencies, and the determined upper-state energy term values are presented in Table 1.

### The observed infrared transitions and assignments of CH_3_NH_2_

In Table 1, the transition notation of *P*/*Q*/*R*(*υ*_*t*_ *S*_*t−i*_ *K, J*) expresses the assigned quantum numbers for each of the identified spectral lines belonging to the *P*-, *Q*-, and *R*-branch, respectively. Here, *υ*_*t*_ is the torsional quantum number, *S*_*t-i*_ is the torsion-inversion symmetry (*A* or *E* for torsional species and *a* or *s* for inversion species), and *K* is the projection along the molecular near-symmetry *a*-axis of the rotational angular momentum *J*. For asymmetry *K*-doubling levels of *A* torsional symmetry, we add a superscript + or − to *K* to indicate the resolved doublet components. The C−N stretching fundamental is a parallel *a*-type band, the transition selection rules are Δ*υ*_t_ = 0, Δ*K* = 0, and Δ*J* = 0, ±1. Other researchers^22^ use another common notation of the *G*_12_ or *D*_3h_ molecular symmetry group species *A, B, E*_1_, and *E*_2_, which corresponds here to *As, Aa, Ea*, and *Es*, respectively. The second and third columns list the determined transition frequencies in MHz and in wavenumbers, respectively, according to the specific CO_2_-microwave sideband used. For example, the Lamb-dip signals I and II in Fig. 2 observed with the lower microwave sideband of the 9*P*22 CO_2_ laser line are assigned as transition *Q*(0 *As* 8, 8) at a transition frequency of 1044.593391 cm^−1^ and as *Q*(0 *As* 5, 5) at 1044.592375 cm^−1^, respectively. The latter was first observed and reported in our previous work^28^, and has been confirmed here. In Table 1, a letter U in the first column denotes a line that is still unassigned in the spectrum. As lines of *s* inversion symmetry are weak with only 1/3 the intensity of the corresponding *a* lines due to the relative spin statistical weights, our data are primarily for the *a* inversion species.

**Figure 4:**
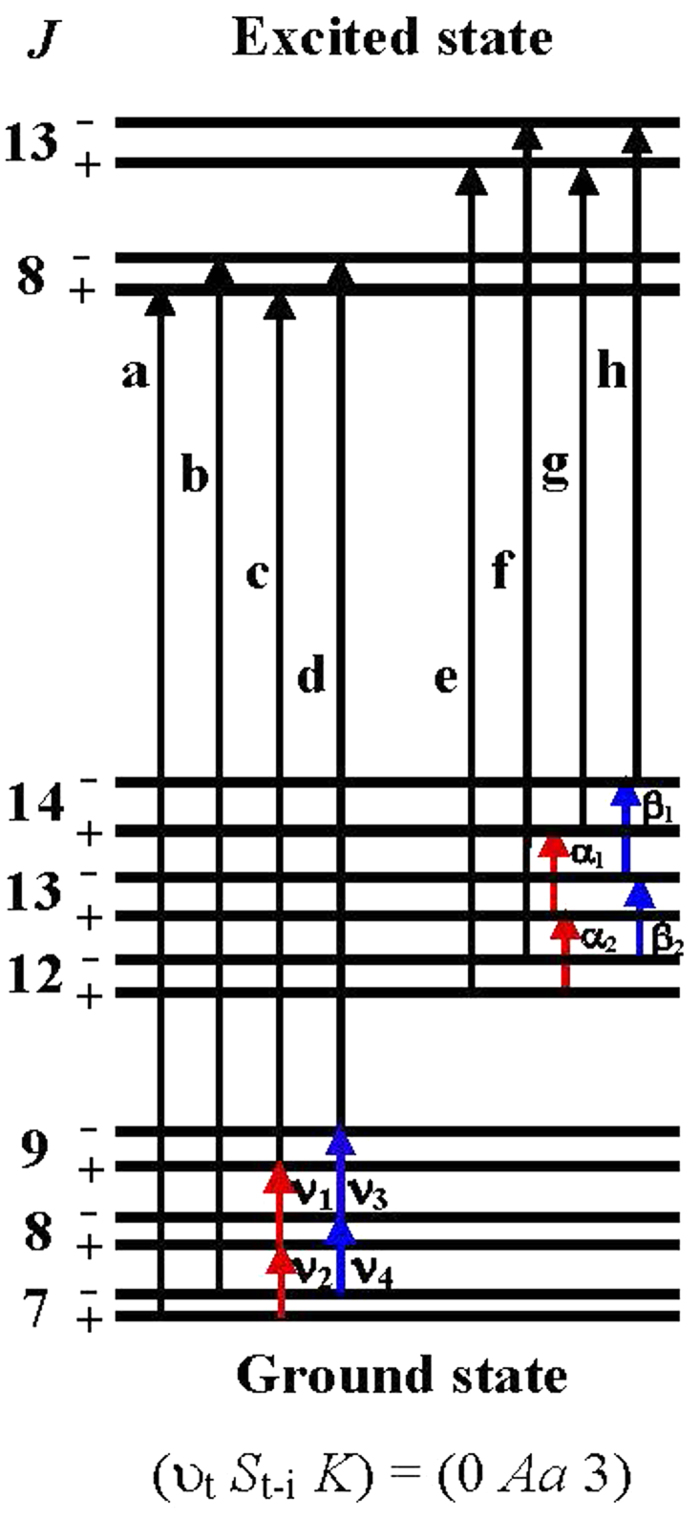
*K*−doublet energy-level diagram for the observed 8 infrared transitions accessing the (*υ*_*t*_ *S*_*t − i*_ *K*)  = (0 *Aa* 3) substate of CH_3_NH_2_ in the Lamb-dip sub-Doppler spectrum. The measured infrared transition frequencies have been given in Table 1. The calculated frequencies (in MHz) for ground-state transitions in Ref. 17 are ν_1_ = 399010.809, ν_2_ = 354681.521, ν_3_ = 399027.383, ν_4_ = 354690.570. The measured transition frequencies (in MHz) in the ground states in Ref. 17 are α_1_ = 620591.113, α_2_ = 576287.579, β_1_ = 620746.167, β_2_ = 576394.504. The asymmetry splittings of the energy levels are exaggerated for clarity.

For a substantial number of transitions, we could test our assignments and measurement accuracy via application of the Rydberg-Ritz combination principle to closed loops involving the observed lines. As an example for illustration, an energy-level diagram for the (*υ*_*t*_ *S*_*t−i*_ *K*) = (0 *Aa* 3) sub-band is shown in Fig. 4. For each pair of transitions sharing a common upper-state level in Fig. 4, we can form four closed loops involving the eight infrared transitions with labels *a* to *h* and the eight microwave transitions in the ground state. By using the predicted microwave frequencies^17^ (uncertainty of 0.06 MHz) in Loops 1 and 2 and the observed microwave frequencies^17^ (uncertainty of 0.06 MHz) in Loops 3 and 4, we calculate the loop closure defects (in MHz) as follows:

Loop 1: δ_8_^+^ = a − c − ν_1_ − ν_2_ = (9*P*10 + 14334.992) − (9*P*36 − 15429.241) − 399010.809 − 354681.521 = − 0.14,

Loop 2: δ_8_^−^ = b − d − ν_3_ − ν_4_ = (9*P*10 + 14339.825) − (9*P*36 − 15450.205) − 399027.383 − 354690.570 = 0.01,

Loop 3: δ_13_^+^ = e − g − α_1_ − α_2_ = (9*P*2 + 13882.574) − (9*P*44 − 14507.345) − 620591.113 − 576287.579 = 0.01,

Loop 4: δ_13_^−^ = f − h − β_1_ − β_2_ = (9*P*2 + 13914.907) − (9*P*44 − 14736.996) − 620746.167 − 576394.504 = − 0.01.

Since each loop contains two infrared transitions, the fact that these loop defects are all so close to zero confirms the transition assignments in each of the loops and our experimental uncertainty of 0.1 MHz. Over 30 closed combination loops of transitions have been formed from the present and previous sub-Doppler observations. Line assignments shown with a letter L in Table 1 are confirmed from these frequency combination closure relations. Such calculations of loop defects are very useful for providing secure assignments for the resolved *K*-doublet lines and for the component lines in blended features, especially for those spectral lines located around the crowded band center. We noticed that when term values for the ground state (kindly provided by N. Ohashi from his microwave and FIR analyses^7^,^14^) are used to calculate the related energy differences involved in the above mentioned loops, we found that the loop defects in MHz from Loop 1 to Loop 4 are −1.05, −2.25, −0.72, and −2.58, respectively. This indicates the estimated uncertainty of those term values to be about 1 MHz, consistent with the estimate from the microwave experimental study by Ilyushin *et al*.^9^.

### Comparison of the measured transition frequencies with those in the FT spectrum

**Figure 5:**
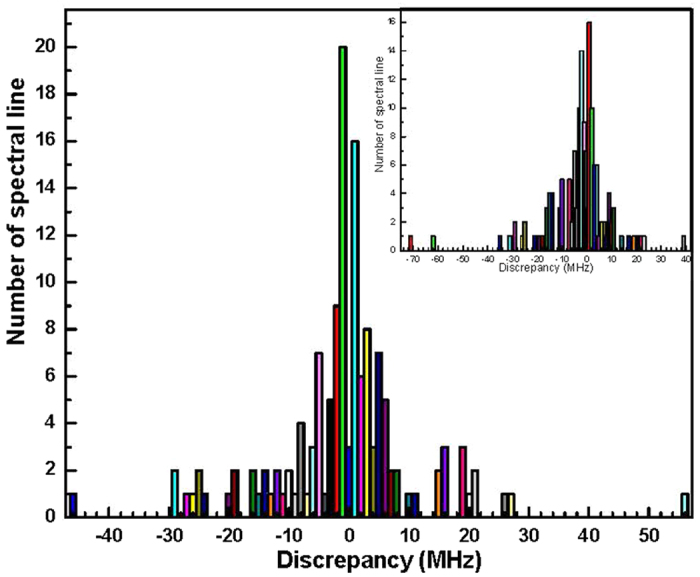
Histogram of the deviations (in MHz) for transition frequencies between our Lamb-dip measurements presented in Table 1 and the FT infrared data reported in Ref. 22. The inset shows the histogram for the FT infrared data reported in Ref. 21.

The last two columns of Table 1 show the frequency differences between the present Lamb-dip measurements and the data in the FT spectrum reported in Ref. 21 and Ref. 22, respectively. A blank space indicates either a new assignment or an unidentified U line. A histogram illustrating the distribution of these differences is given in Fig. 5. The inset shows the histogram for the FT infrared data reported in Ref. 21. We see that most of the deviations are less than several megahertz, but some of them are tens of megahertz. The mean value of the absolute deviation is about 5 MHz and 8 MHz for the FT data in Ref. 21 and Ref. 22, respectively, which reflects the frequency accuracy of the FT data estimated in these two works for infrared transitions in the C−N stretching band of CH_3_NH_2_.

### Power-series expansions of the energy term values in the excited state

The fourth column of Table 1 lists energy term values *W*(*υ*_*t*_ *S*_*t* − *i*_ *K, J*) for the upper levels of the corresponding transitions in the first column, obtained by adding our measured transition frequencies in wavenumbers to the Ohashi calculated ground-state energies, referenced to the (*υ*_*t*_ *S*_*t−i*_ *K, J*) = (0 *As* 0, 0) ground level as the energy zero. When two or three transitions were observed having a common upper level but giving independent values, the average of those term values was taken. In order to determine the *J*-independent origins of 27 C−N stretching substates, their term values were least-squares fitted to *J*(*J*+1) power-series expansions


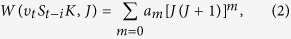


where *a*_0_ is the *J*-independent substate origin, *a*_1_ is an effective rotational constant, and *a*_2_ and *a*_3_ are effective centrifugal distortion constants. The obtained expansion coefficients *a*_0_, *a*_1_, *a*_2_, and *a*_3_ for 27 substates are shown in Table 2, in which the 1-*σ* standard deviations are in units of the last quoted digit.

### Asymmetry splittings and asymmetry-splitting constants of *A* symmetry levels in the excited state

A rotational level with *A* symmetry of CH_3_NH_2_ may split due to the higher-order vibration-rotation interactions. For the resolved *K-*doublet lines, the transition selection rules are *K*^±^ ↔ *K*^±^ for Δ*J* = ±1 and *K*^±^ ↔ *K* ^∓^ for Δ*J* = 0. We have observed 57 Lamb-dip signals for resolved *K*-doublet lines resulting from the asymmetry splittings and precisely determined their transition frequencies. From the calculated level splittings in the ground state, asymmetry splittings for *Aa* levels with *K* = 1, 2, and 3 in the *υ*_*t*_ = 0 excited state have been determined and are shown in Table 3. The observed asymmetry splittings Δ*E*(*υ*_t_ *S*_t−i_ *K, J*) can be approximately represented by


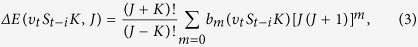


where *b*_0_ is the principal asymmetry splitting constant and other *b*_m_ with *m* ≥ 1 are higher-order corrections. The resulting series expansion coefficients from least-squares fits of the observed asymmetry splittings to Eq. (3) for the three *K*-states are presented in Table 3.

## Conclusion

In this work for a molecule with the two strongly coupled large-amplitude internal motions of torsion and inversion, by using microwave sidebands of CO_2_ laser lines as frequency-tunable infrared sources in a sub-Doppler spectrometer, Lamb-dip spectroscopy of the C−N stretching band of CH_3_NH_2_ has been systematically studied. Many blended features and unresolved *K*-doublet lines involving wide variations in relative intensities in dense Doppler-limited spectra have been separated at high resolution. More than 50 *K*-doublet lines have been observed and the asymmetry-splitting constants for levels with *K* = 1, 2, and 3 in the excited state have been determined. Over 150 transitions have been assigned and identified into 27 substates and their transition frequencies have been precisely measured with an absolute accuracy of ±0.1 MHz. Energy term values for the upper levels of these assigned transitions have been determined and have been fitted to *J*(*J* + 1) power-series expansions for each substate to determine the *J*-independent C−N stretching substate origins and effective rotational constants. The Rydberg-Ritz combination principle was fully used in calculations of the defects for closed combination loops involving our observed transitions for confirming, revising and extending the transition assignments from previously reported results. Our experiment demonstrates the capabilities of the current experimental setup to precisely study the sub-Doppler infrared spectroscopies of isotopic species of CH_3_NH_2_ and of molecules with more than two internal motions. Our results constitute a high-accuracy database for frequency calibration in the 9–11 *μ*m region, provide more accurate spectral information for excited vibrational states to clearly map the rich and complex energy structures, to reveal the complex interaction mechanisms relevant to the C−N stretching of CH_3_NH_2_, to support further possible astronomical detections of interstellar CH_3_NH_2_, and to assign more energy levels and transition systems for optically pumped far-infrared laser emissions of CH_3_NH_2_.

## Methods

The tunable microwave radiation is generated by a microwave synthesizer and is amplified by a traveling-wave-tube amplifier to a power of about 20W before being fed to the modulator. Under this condition, the typical conversion efficiency into the sidebands from the incident laser beam is about 0.8%. In order to saturate the transitions of CH_3_NH_2_ at low pressures of several mTorr in this work, the full output radiation from the modulator is focused into our multi-reflection absorption cell with a total absorption path of 9.6 m in 16 transits. For observation of the saturation Lamb-dips by generating a counter-propagating beam inside the cell, a mirror M5 is placed at the exit of the cell window to reflect the radiation which has passed through the absorption cell back into the cell. This mirror is adjusted carefully so that the retro-reflected radiation nearly coincides with the incoming radiation but is slightly shifted and passes through a tunable Fabry–Pérot etalon filter for selecting only the desired sideband containing the CH_3_NH_2_ absorption signal. In such an optical arrangement, only those molecules moving at zero velocity parallel to the beam can absorb both the two counter-propagating laser radiations which have the same frequency, creating a saturation dip at the center of the absorption curve. This narrow dip is then detected by a liquid-N_2_-cooled HgCdTe detector as a Lamb-dip signal. In order to increase the probing sensitivity for the spectral signals, we use 1 kHz modulation of the sideband laser frequency for source modulation and demodulate the detected signal with a digital lock-in amplifier operating in the second-derivative (2*f*) detection mode to display the spectral lines. The signals are then sent to a computer for recording and analysis. A commercial CH_3_NH_2_ sample supplied by BOC Specialty Gases with a stated purity of 99.5% was used in the experiment without further purification. High attention should be taken for avoiding the possible confusion in the spectral analysis from NH_3_ impurity lines.

## Additional Information

**How to cite this article**: Sun, Z.-D. *et al*. Lamb-dip spectroscopy of the C − N stretching band of methylamine by using frequency-tunable microwave sidebands of CO_2_ laser lines. *Sci. Rep.* **6**, 34270; doi: 10.1038/srep34270 (2016).

## Figures and Tables

**Table 1:** Observed infrared transitions and their upper-state term values for the C−N stretching band of CH_3_NH_2_.

aTransition *R*/*P*/*Q*(υ_t_ *S*_t−i_ *K, J*)	bCO_2_ laser line ± microwave req. (MHz)	cν_obs_. (cm^−1^)	Upper-state term value (cm^−1^)	dν_FT1_ - ν_obs._ (MHz)	eν_FT2_ - ν_obs._ (MHz)
*R*(0 *Ea* 4, 23)	9*R*16 − 11898.876	1075.590916	1527.054716	−29.3	−26.3
*R*(0 *Aa* 1^−^, 23)	9*R*16 − 12671.411	1075.565147	1489.380347	−15.5	−14.7
*R*(0 *Ea* 4, 20)	9*R*10 + 10911.802	1072.247745	1426.195845	−13.9	−15.4
*R*(0 Ea 7, 20)	9*R*10 + 10894.123	1072.247155	1515.100055	3.8	
*R*(0 *Aa* 6, 20)	9*R*10 + 10689.436	1072.240327	1480.052327	1.3	−1.0
*R*(0 *Aa* 5, 20)	9*R*10 + 10553.581	1072.235796	1450.347096	−61.6	−45.4
*R*(0 *Aa* 3^+^, 20)	9*R*10 + 10507.877	1072.234271	1407.452171	−15.9	0.3
*R*(0 *Aa* 1^−^, 20)	9*R*10 + 10487.973	1072.233607	1388.031807	4.0	20.2
*R*(0 *Aa* 3^−^, 20)	9*R*10 + 10453.378	1072.232453	1407.567753	38.6	54.8
*R*(0 *Ea* −2, 20)	9*R*10 + 10305.241	1072.227512	1393.266612	−9.6	−6.7
*R*(0 *Aa* 2^−^, 18)	9*R*8 − 8791.434	1070.169057	1333.969857	−1.7	2.7
*R*(0 *Aa* 2^+^, 18)	9*R*8 − 9899.311	1070.132103	1334.975403	−1.8	−4.0
*R*(0 *Ea* −3, 18)	9*R*8 − 11187.383	1070.089137	1347.673137	9.4	6.9
*R*(0 *Ea* 3, 18)	9*R*8 − 11829.536	1070.067717	1347.269817	6.4	18.5
*R*(0 *Aa* 1^+^, 18)	9*R*8 − 15025.886	1069.961098	1322.810798	1.6	0.5
*R*(0 *Aa* 6, 18)	9*R*8 − 15611.927	1069.941550	1420.150150	−14.7	−25.5
*R*(0 *Aa* 3^−^, 18)	9*R*8 − 15931.388	1069.930894	1347.569494	−15.7	−18.4
*R*(0 *Ea* −2, 18)	9*R*8 − 16465.53	1069.913077	1333.443077	−3.5	0.3
*R*(0 *Ea* 2, 18)	9*R*8 − 16694.656	1069.905434	1334.541634	3.8	0.8
*R*(0 *Aa* 7, 18)	9*R*6 + 11061.409	1069.383062	1454.799162	6.0	−0.7
*R*(0 *Aa* 4^+^, 18)	9*R*6 + 10912.404	1069.378092	1365.464792	−6.9	−8.3
*R*(0 *Aa* 4^−^, 18)	9*R*6 + 10896.661	1069.377566	1365.462566	8.8	7.4
*R*(0 *Ea* −1, 17)	9*R*6 − 8350.046	1068.735565	1295.477665	16.7	
*R*(0 *Aa* 4^−^, 16)	9*R*4 − 15504.517	1067.021935	1311.376435	8.6	4.1
*R*(0 *Aa* 7, 16)	9*R*4 − 16034.697	1067.004250	1400.722550	−3.2	−0.5
*R*(0 *Aa* 2^+^, 15)	9*R*2 + 15797.556	1066.564310	1255.648210		
*R*(0 *Aa* 2^−^, 15)	9*R*2 + 15795.090	1066.564227	1255.115127	−0.7	2.1
*R*(0 *Ea* −5, 15)	9*R*2 + 13606.873	1066.491236	1311.601836	0.8	0.5
*R*(0 *Ea* −3, 15)	9*R*2 + 12983.316	1066.470437	1268.633737	1.1	0.6
*R*(0 *Aa* 8, 15)	9*R*2 + 11893.876	1066.434097	1416.913897	−25.6	
*R*(0 *Ea* 3, 15)	9*R*2 + 11845.906	1066.432497	1268.214197	22.3	19.9
*R*(0 *Ea* 1, 15)L	9*R*2 + 10302.783	1066.381024	1248.412624	2.3	−0.2
*R*(0 *Aa* 6, 15)	9*R*2 + 10069.842	1066.373254	1341.234754	11.0	20.7
*R*(0 *Aa* 3^−^, 15)	9*R*2 + 9488.821	1066.353873	1268.554973	−31.2	−28.9
*R*(0 *Aa* 5, 15)	9*R*2 + 9277.964	1066.346840	1311.482840	−6.5	−4.5
*R*(0 *Ea* −2, 15)	9*R*2 + 8780.626	1066.330250	1254.592450	−24.5	−24.2
*R*(0 *Ea* −5, 12)	9*P*2 + 17768.953	1062.758673	1245.792673	−5.1	−7.1
*R*(0 *Ea* −3, 12)L	9*P*2 + 16567.900	1062.718611	1202.770811	4.3	−1.7
*R*(0 *Aa* 8, 12)	9*P*2 + 16467.650	1062.715267	1351.145967	2.9	5.2
*R*(0 *Ea* 1, 12)	9*P*2 + 15260.579	1062.675003	1182.112603	0.9	−0.3
*R*(0 *Aa* 1^−^, 12)L	9*P*2 + 14932.119	1062.664047	1181.933847	−15.4	−15.5
*R*(0 *Ea* 4, 12)L	9*P*2 + 14173.561	1062.638744	1221.484244	−2.7	−1.2
*R*(0 *Ea* 5, 12)	9*P*2 + 13932.828	1062.630714	1245.862714	−29.1	
*R*(0 *Aa* 3^−^, 12)L	9*P*2 + 13914.907	1062.630116	1202.713316	−11.2	−13.7
*R*(0 *Aa* 3^+^, 12)L	9*P*2 + 13882.574	1062.629038	1202.706238	21.1	18.6
*R*(0 *Aa* 5, 12)	9*P*2 + 13705.152	1062.623120	1245.683420	−2.3	0.7
*R*(0 *Ea* −2, 12)L	9*P*2 + 13373.523	1062.612058	1188.822658	−6.7	−4.7
U	9*P*2 + 13332.331	1062.610684			
*R*(0 *Ea* 2, 12)	9*P*2 + 12741.567	1062.590978	1189.173578	−9.7	−5.7
*R*(0 *Ea* −1, 12)	9*P*2 + 12730.848	1062.590620	1179.746320	1.0	5.1
*R*(0 *Aa* 1^+^, 12)	9*P*2 + 11318.384	1062.543506	1179.529006	−3.7	−0.2
*R*(0 *Aa* 4, 11)	9*P*4 + 8971.098	1060.869910	1201.743110	−4.7	−0.1
*R*(0 *Aa* 1^+^, 9)L	9*P*6 − 8477.145	1058.665947	1127.387047	−3.1	0.7
*R*(0 *Aa* 2^+^, 8)	9*P*8 + 9991.249	1057.633433	1122.155833	−4.7	−0.7
*R*(0 *Aa* 2^−^, 8)	9*P*8 + 9671.323	1057.622762	1122.096162	−3.2	−2.1
*R*(0 *Aa* 4, 8)	9*P*8 − 9088.952	1056.996986	1153.512786	−1.5	0.1
*R*(0 *Ea* 1, 7)	9*P*10 + 16357.707	1056.170702	1101.016902	−0.5	2.2
*R*(0 *Ea* −3, 7)L	9*P*10 + 16082.288	1056.161515	1122.270215	−1.5	−5.9
*R*(0 *Aa* 6^−^, 7)	9*P*10 + 15748.817	1056.150392	1195.035492		
*R*(0 *Aa* 6^+^, 7)	9*P*10 + 15748.072	1056.150367	1195.035467	−14.7	−12.4
*R*(0 *Aa* 1^−^, 7)	9*P*10 + 15431.349	1056.139802	1100.808202	−6.8	−4.1
*R*(0 *Aa* 3^−^, 7)L	9*P*10 + 14339.825	1056.103393	1122.240693	−7.4	−7.5
*R*(0 *Aa* 3^+^, 7)L	9*P*10 + 14334.992	1056.103232	1122.240332	−2.6	−2.7
*R*(0 *Aa* 5^−^, 7)	9*P*10 + 14226.358	1056.099608	1165.243108		
*R*(0 *Aa* 5^+^, 7)	9*P*10 + 14224.595	1056.099549	1165.243049	−10.6	−7.4
*R*(0 *Ea* 3, 7)L	9*P*10 + 14191.512	1056.098446	1121.821546	22.5	25.6
*R*(0 *Ea* −2, 7)L	9*P*10 + 13868.159	1056.087660	1108.348360	1.3	−0.9
*R*(0 *Ea* 2, 7)L	9*P*10 + 12586.227	1056.044899	1108.598499	1.7	−1.8
*R*(0 *Aa* 1^+^, 7)L	9*P*10 + 11458.187	1056.007272	1099.853072	−0.5	−0.1
*R*(0 *Ea* −1, 7)L	9*P*10 + 11298.615	1056.001949	1100.011549	2.6	4.6
*R*(0 *Aa* 4, 6)	9*P*12 + 12380.893	1054.336485	1128.671685	−5.4	−5.4
*R*(0 *Ea* 1, 5)L	9*P*12 − 14021.903	1053.455783	1078.900283	0.7	1.2
*R*(0 *Aa* 1^−^, 5)L	9*P*12 − 15401.199	1053.409774	1078.668574	−5.6	−1.9
*R*(0 *Aa* 3, 5)	9*P*12 − 16245.122	1053.381624	1100.293124	−1.4	−3.2
*R*(0 *Ea* 3, 5)	9*P*12 − 16712.009	1053.366050	1099.863150	11.3	10.2
*R*(0 *Ea* −2, 5)	9*P*12 − 16817.968	1053.362516	1086.392416	5.4	4.2
*R*(0 *Ea* 2, 5)	9*P*12 − 18065.890	1053.320890	1086.642890	−3.4	3.2
*Q*(0 *Aa* 3^+^, 4)	9*P*22 − 10210.545	1044.681083	1084.198083	2.3	
*Q*(0 *As* 8, 8)	9*P*22 − 12839.477	1044.593391	1270.813991	−17.5	−19.6
*Q*(0 *Ea* −6, 6)	9*P*22 − 15577.846	1044.502049	1172.979549	−5.4	
*Q*(0 *Ea* 9, 9)	9*P*22 − 16084.970	1044.485133	1330.001133	2.0	5.6
U	9*P*22 − 17775.882	1044.428730			
U	9*P*24 + 17848.480	1043.758600			
*Q*(0 *Ea* −8, 13)	9*P*24 + 17816.997	1043.757550	1351.457050	6.9	7.0
*Q*(0 *Ea* 9, 13)	9*P*24 + 17809.443	1043.757298	1397.236398	14.4	14.6
*Q*(0 *Aa* 11, 13)	9*P*24 + 16894.708	1043.726786	1505.045186	−2.3	−2.2
*Q*(0 *Aa* 8, 12)	9*P*24 + 16465.533	1043.712470	1332.143170	−0.3	-0.5
*Q*(0 *Aa* 7, 8)	9*P*24 + 16151.914	1043.702009	1229.649909	−5.1	−0.2
*Q*(0 *Aa* 6, 12)	9*P*24+14714.233	1043.654053	1256.44835	−0.1	1.0
*Q*(0 *Aa* 9, 12)	9*P*24 + 14041.047	1043.631598	1377.577598	−2.7	
*Q*(0 *Aa* 7, 9)	9*P*24 + 11963.481	1043.562298	1242.813298	3.0	6.5
U	9*P*24 + 10809.657	1043.523810			
*Q*(0 *Aa* 11, 14)	9*P*24 + 10233.275	1043.504584	1525.496884	−10.1	−8.6
*P*(0 *Aa* 1^+^, 6)	9*P*32 + 10145.824	1035.812044	1069.412640	0.8	0.3
*P*(0 *Ea* 1, 7)L	9*P*34 + 16971.825	1034.054118	1078.900320	2.5	5.0
*P*(0 *Aa* 1^−^, 7)L	9*P*34 + 15354.390	1034.000166	1078.668566	2.3	1.7
*P*(0 *Aa* 2^−^, 9)	9*P*36 − 9648.259	1031.155600	1108.931200	−0.8	0
*P*(0 *Aa* 1^+^, 9)L	9*P*36 − 10356.138	1031.131987	1099.853087	1.7	0.4
*P*(0 *Ea* −1, 9)L	9*P*36 − 10624.323	1031.123042	1100.011642	−1.8	−0.4
*P*(0 *Aa* 2^+^, 9)	9*P*36 − 10809.311	1031.116871	1108.969271	−0.8	0.9
*P*(0 *Ea* −3, 9)	9*P*36 − 13698.547	1031.020497	1122.270197	−5.8	−11.9
*P*(0 *As* 3, 9)	9*P*36 − 14350.507	1030.998750	1122.176450	−3.2	−1.7
*P*(0 *Ea* 4, 9)	9*P*36 − 14830.98	1030.982723	1141.037623	−9.9	−10.6
*P*(0 *Aa* 3^+^, 9)	9*P*36 − 15429.241	1030.962767	1122.240367	−19.0	−11.6
*P*(0 *Aa* 3^−^, 9)	9*P*36 − 15450.205	1030.962068	1122.240768	2.0	9.4
*P*(0 *Ea* 3, 9)L	9*P*36 − 15604.449	1030.956923	1121.821623	−0.3	−0.4
*P*(0 *Ea* −2, 9)L	9*P*36 − 16137.042	1030.939157	1108.348360	3.2	1.1
*P*(0 *Ea* 2, 9)L	9*P*36 − 17653.154	1030.888585	1108.598490	4.1	2.7
*P*(0 *Aa* 1^−^, 10)	9*P*38 − 9484.598	1029.125720	1114.089120	−2.0	1.3
*P*(0 *Aa* 4, 10)	9*P*38 − 16148.642	1028.903431	1153.512831	0.7	0
*P*(0 *Ea* −1, 11)	9*P*40 + 17092.541	1027.952318	1127.560120	1.4	4.1
*P*(0 *Aa* 1^+^, 11)	9*P*40 + 16989.214	1027.948871	1127.387171	−0.2	2.1
*P*(0 *Aa* 2^−^, 11)	9*P*40 + 15993.048	1027.915643	1136.722040	0.9	2.8
*P*(0 *Aa* 2^+^, 11)	9*P*40 + 13721.694	1027.839878	1136.811378	11.0	14.0
*P*(0 *Ea* 4, 11)	9*P*40 + 10281.031	1027.725110	1168.829210	7.0	4.5
*P*(0 *Aa* 5, 11)	9*P*40 + 9887.452	1027.711982	1193.033982	9.9	18.7
*P*(0 *Aa* 3^+^, 11)	9*P*40 + 9730.095	1027.706733	1150.039030	−15.0	−18.6
*P*(0 *Ea* −2, 11)	9*P*40 + 9187.589	1027.688637	1136.157337	2.3	3.7
*P*(0 *Ea* 1, 12)	9*P*42 + 15646.148	1025.819764	1145.257364	−2.1	0.1
*P*(0 *Aa* 1^−^, 12)	9*P*42 + 15032.225	1025.799286	1145.069086	0.2	−0.5
*P*(0 *Ea* −1, 13)	9*P*42 − 16840.998	1024.736110	1160.897510	−1.6	−1.1
*P*(0 *Aa* 1^+^, 13)	9*P*42 − 17569.495	1024.711810	1160.702410	−1.6	−1.1
*P*(0 *Ea* −3, 14)L	9*P*44 − 11971.706	1022.790042	1202.770842		
*P*(0 *Aa* 2^+^, 14)	9*P*44 − 12189.339	1022.782782	1189.601080	−2.6	−4
*P*(0 *Aa* 6, 14)	9*P*44 − 12928.003	1022.758143	1275.454543	1.0	2.1
*P*(0 *Ea* 3, 14)	9*P*44 − 13533.332	1022.737952	1202.336550	−1.7	−0.8
*P*(0 *Ea* 4, 14)L	9*P*44 − 13960.687	1022.723697	1221.484200	−9.6	−9.5
*P*(0 *Ea* −2, 14)L	9*P*44 − 14254.030	1022.713912	1188.822610	7.9	26.0
*P*(0 *Aa* 3^+^, 14)L	9*P*44 − 14507.345	1022.705462	1202.706262	−2.3	−4.9
*P*(0 *Aa* 3^−^, 14)L	9*P*44 − 14736.996	1022.697802	1202.713402	0.7	−0.8
*P*(0 *Ea* −1, 15)	9*P*46 + 14919.509	1021.554573	1200.096773	−3.2	
*P*(0 *Aa* 1^+^, 15)	9*P*46 + 11162.841	1021.429264	1199.799864	−5.3	−2.1
*P*(0 *Aa* 1^−^, 15)	9*P*46 − 10682.479	1020.700583	1202.570383	1.6	−0.1
*P*(0 *Aa* 4, 15)	9*P*46 − 16527.760	1020.505605	1241.210105	−3.9	−4.9
*P*(0 *Ea* −3, 16)	9*P*48 + 14709.120	1019.391336	1245.215836	0	−1.9
*P*(0 *Ea* 3, 16)	9*P*48 + 13389.423	1019.347316	1244.790816	−34.8	−28.5
*P*(0 *Ea* 7, 16)	9P48 + 13353.259	1019.346110	1352.910110	1.4	
*P*(0 *Aa* 6, 16)	9*P*48 + 13345.498	1019.345851	1317.848051	9.2	15.4
*P*(0 *Ea* −2, 16)	9*P*48 + 12891.933	1019.330722	1231.214822	0.2	0.6
*P*(0 *Aa* 2^+^, 16)	9*P*48 + 12421.741	1019.315038	1232.157838	−1.4	−2.7
*P*(0 *Ea* 4, 16)	9*P*48 + 12335.332	1019.312155	1263.896255	−21.0	−24.5
*P*(0 *Ea* 5, 16)	9*P*48 + 12295.030	1019.310811	1288.263811	19.3	15.8
*P*(0 *Aa* 5, 16)	9*P*48 + 12229.499	1019.308625	1288.089125	−2.4	3.0
*P*(0 *Aa* 3^+^, 16)	9*P*48 + 11781.408	1019.293678	1245.128078	0.2	4.6
*P*(0 *Aa* 3^−^, 16)	9*P*48 + 11308.036	1019.277888	1245.144388	−0.7	0.2
*P*(0 *Ea* 2, 17)	9*P*50 + 17961.451	1017.320072	1255.207572	−1.3	0
*P*(0 *Ea* 1, 17)L	9*P*50 + 15685.814	1017.244164	1248.412664	1.3	0.8
*P*(0 *Aa* 1^−^, 17)	9*P*50 + 15357.100	1017.233200	1248.244800	−11.2	−7.4
*P*(0 *Aa* 4, 17)	9*P*50 + 9686.673	1017.044055	1286.526755	−24.8	−23.6
*P*(0 *Aa* 2^−^, 18)	9*P*50 − 17254.659	1016.145389	1279.946189	3.9	1.5
*P*(0 *Ea* −2, 19)	9*P*52 − 10876.861	1014.155075	1305.707275	2.5	5.5
*P*(0 *Aa* 6, 19)	9*P*52 − 12143.902	1014.112811	1392.386211	−0.1	−0.4
*P*(0 *Ea* −6, 19)	9*P*52 − 12921.993	1014.086857	1392.296957	−70.7	
*P*(0 *Aa* 5, 19)	9*P*52 − 13002.243	1014.084180	1362.650680	9.5	15.1
*P*(0 *Aa* 3^−^, 19)	9*P*52 − 14888.525	1014.021260	1319.767360	−0.4	−0.6
*P*(0 *Aa* 2^+^, 19)	9*P*52 − 16384.082	1013.971374	1307.056674	1.0	2.5

^a^Letter L indicates that the line assignment has been confirmed from a frequency combination loop; a letter U denotes an unassigned line.

^b^Observed transition frequencies in MHz are expressed by the frequency of the CO_2_ laser line and the determined microwave frequency from the fitting to the recorded Lamb-dip signal.

^c^Observed transition frequencies are converted into wavenumbers using a factor of 29979.2458 MHz/cm^−1^ and CO_2_ laser frequencies from Ref. 30.

^d^Differences (in MHz) between the observed transition frequencies in the present work and values from the FT infrared wavenumbers reported in Ref. 21. A blank space indicates either a new assignment or an unidentified U line to the data in Ref. 21.

^e^Differences (in MHz) between the observed transition frequencies in the present work and values from the FT infrared wavenumbers reported in Ref. 22. A blank space indicates either a new assignment or an unidentified U line to the data in Ref. 22.

**Table 2:** *J*(*J* + 1) power-series expansion coefficients (in cm^−1^) of upper-state term values in 27 substates of the C−N stretching band of CH_3_NH_2_.

Substate (*υ*_*t*_ *S*_*t−i*_ *K*)	*a*_0_	*a*_1_	*a*_2_ × 10^6^	*a*_3_ × 10^10^
(0 *Aa* 0)*	1044.912526(461)	0.732866(11)	−10.72(8)	44.1(16)
(0 *Aa* 1^+^)	1047.66065(167)	0.725175(4)	−3.92(26)	27.3(4)
(0 *Aa* 1^−^)	1047.662408(196)	0.738380(3)	−3.34(1)	−5.0(1)
(0 *Aa* 2^+^)	1056.257642(711)	0.731710(12)	5.73(6)	−29.0(9)
(0 *Aa* 2^−^)	1056.254285(389)	0.731812(7)	−2.63(3)	1.1(4)
(0 *Aa* 3^+^)	1069.565927(15)	0.731614(0)	−0.31(1)	−5.5(0)
(0 *Aa* 3^−^)	1069.566059(76)	0.731610(1)	−0.28(1)	5.9(1)
(0 *Aa* 4)	1087.76225(210)	0.730430(40)	−1.69(20)	−0.2(3)
(0 *Aa* 5)	1112.580226(61)	0.731490(1)	−0.84(0)	−0.2(0)
(0 *Aa* 6)	1142.383547(391)	0.731342(7)	−1.01(3)	0.4(2)
(0 *Aa* 7)	1176.989660(29)	0.7314660(0)	−1.02(0)	0
(0 *Aa* 8)	1218.14342(465)	0.730775(22)		
(0 *Aa* 11)	1372.109149(0)	0.730418(0)		
(0 *Ea* 0)*	1045.101654(472)	0.731802(12)	−10.24(6)	34.2(9)
(0 *Ea* 1)	1047.93850(858)	0.737074(237)	2.77(18)	−107(37)
(0 *Ea* 2)	1055.93689(927)	0.730844(209)	7.42(11)	−34(17)
(0 *Ea* 3)	1069.121205(380)	0.731947(11)	78(7)	1.4(0)
(0 *Ea* 4)	1088.374933(240)	0.731462(4)	-0.49(2)	−9(2)
(0 *Ea* 5)	1112.77336(123)	0.731406(29)	−0.8(1)	0
(0 *Ea* 6)	1142.535800(365)	0.731324(6)		
(0 *Ea* 7)	1177.49078(396)	0.730786(131)		
(0 *Ea* 9)	1264.227504(0)	0.730818(0)		
(0 *Ea* −1)	1047.6819(828)	0.727080(889)	−7(2)	0
(0 *Ea* -2)	1055.62915(546)	0.732554(97)	−4.2(4)	10(5)
(0 *Ea* −3)	1069.575107(628)	0.731907(12)	−0.49(6)	8.0(1)
(0 *Ea* −5)	1112.674698(42)	0.731554(1)	−0.75(0)	0
(0 *Ea* −6)	1142.275112(0)	0.731058(0)		
(0 *Ea* −7)	1177.492590(0)	0.731449(0)		
(0 *Ea* −8)	1218.416547(0)	0.730994(0)		

^*^First reported in Ref. 28 and confirmed in this work.

Energy of the level (*υ*_*t*_ *S*_*t−i*_ *K, J*) = (0 *As* 0, 0) in the ground state is taken as the zero. Errors in parentheses are one standard deviation in units of the last quoted digit.

**Table 3:** Asymmetry splittings Δ*E*(*υ*_t_ *S*_*t−i*_ *K, J*) and asymmetry-splitting constants *b*_m_ (in MHz) for the resolved *K*-doublet levels of *A* torsional symmetry of CH_3_NH_2_.

*J*	Δ*E*(*υ*_t_ *S*_*t−i*_ *K, J*) (MHz)	aObs. − Calc. (MHz)	*K*	*b*_0_	*b*_1_	*b*_2_	*b*_3_	*b*_4_	*b*_5_
7	28634.077	−0.087	1	496.599352	0.533819	−0.004838			
12	72095.319	−1.861							
15	83058.070	−6.763							
8	1788.892	0.035	2	4.325212	−0.107269	9.44 × 10^−4^	−3.35 × 10^−6^	4.10 × 10^−9^	
9	1141.340	0.068							
11	2678.196	0.214							
15	15981.426	1.268							
18	30145.511	3.581							
7	10.823	0	3	8.77 × 10^−4^	−2.91 × 10^−5^	3.61 × 10^−7^	−2.01 × 10^−9^	5.09 × 10^−12^	−4.72 × 10^−15^
9	12.022	0							
12	212.193	−0.001							
14	214.052	−0.011							
16	488.961	−0.078							
20	3465.061	−1.431							

^a^Asymmetry splittings were calculated from the asymmetry-splitting constants in Table 3.

The *K*-doublet levels are in the excited states with *K* = 1, 2, and 3 and in torsional state *υ*_t_ = 0 with the torsion-inversion symmetry *Aa*.
